# DHPV: a distributed algorithm for large-scale graph partitioning

**DOI:** 10.1186/s40537-020-00357-y

**Published:** 2020-09-16

**Authors:** Wilfried Yves Hamilton Adoni, Tarik Nahhal, Moez Krichen, Abdeltif El byed, Ismail Assayad

**Affiliations:** 1 LIMSAD Laboratory, Faculty of sciences, Hassan II University of Casablanca, Casablanca, Morocco; 2 Faculty of CSIT, Albaha University, Al Bahah, Saudi Arabia; 3 ReDCAD Laboratory, University of Sfax, Sfax, Tunisia; 4 LIMSAD Laboratory, ENSEM, Hassan II University of Casablanca, Casablanca, Morocco

**Keywords:** Big graph, Large-scale networks, k-Partition, Graph partitioning algorithms, Distributed computing, GraphX

## Abstract

Big graphs are part of the movement of “Not Only SQL” databases (also called NoSQL) focusing on the relationships between data, rather than the values themselves. The data is stored in vertices while the edges model the interactions or relationships between these data. They offer flexibility in handling data that is strongly connected to each other. The analysis of a big graph generally involves exploring all of its vertices. Thus, this operation is costly in time and resources because big graphs are generally composed of millions of vertices connected through billions of edges. Consequently, the graph algorithms are expansive compared to the size of the big graph, and are therefore ineffective for data exploration. Thus, partitioning the graph stands out as an efficient and less expensive alternative for exploring a big graph. This technique consists in partitioning the graph into a set of k sub-graphs in order to reduce the complexity of the queries. Nevertheless, it presents many challenges because it is an NP-complete problem. In this article, we present DPHV (Distributed Placement of Hub-Vertices) an efficient parallel and distributed heuristic for large-scale graph partitioning. An application on a real-world graphs demonstrates the feasibility and reliability of our method. The experiments carried on a 10-nodes Spark cluster proved that the proposed methodology achieves significant gain in term of time and outperforms JA-BE-JA, Greedy, DFEP.

## Introduction

Graphs are ubiquitous [1] in engineering sciences because they prove to be a flexible model in the modeling of various complex phenomena emanating from various disciplines [2]: biological, sociological, economic, physical and technological. A great deal of research was dedicated to improving methods of analysis for these networks [3, 4]. Nevertheless, the effectiveness and applicability of these methods are still limited to small networks because of the complexity of exhaustive analysis [3]. The analysis of a complex network is very expansive and consumes a lot of hardware resources because of the NP-completeness of the problem [5, 6].

Large-scale network such as social networks (e.g., Facebook and Twitter) [7, 8], road networks [8–12], brain networks [2], etc. with their heterogeneity allow to analyze a chaotic dynamics or represent a complex phenomenon. They represent numerous exciting challenges related to high performance computing problems, where data scalability, program complexity and robustness hardware configurations play an important role [13]. Solving these problems can contribute to efficiently manage the new trend technologies such as big data (e.g., dataViz), distributed systems (e.g., Hadoop [14] and Spark [15]) or future communication networks (e.g., 5G or IoT). Network analysis is widely used in various domains where experimenting relies on large-scale dataset structured as graph data and each information is stored in a vertex an the edges modelize interactions between vertices [8].

**Fig. 1:**
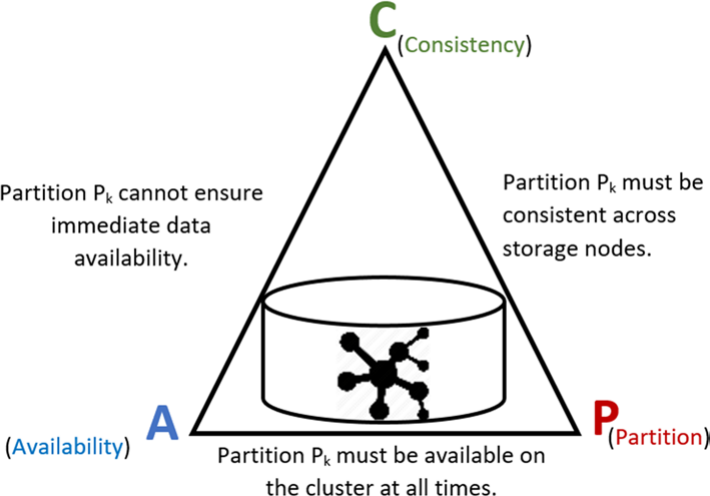
CAP theorem

The analysis of a large-scale network consists of exploring the properties associated with the edges and vertices of a graph. Given a large-scale network, the time complexity of the graph algorithms increases exponentially compared to the number of vertices [1]. Thus, to speed up the performance of graph algorithms, it’s recommended to use distributed system to speed up analytical tasks [16]. This technique is widely used in NoSQL databases. It is effective because compared to the CAP theorem [17], it ensures the consistency and availability of data. Graph-oriented databases cannot guarantee all of the properties of the CAP theorem [18]. Figure 1 shows that the partitioning of a graph ensures only the properties CP and CA of the CAP theorem:

- *Consistency and Availability (CA)* Since the graph data is stored on a distributed system, we cannot guarantee the availability and the consistency of the dataset across the cluster at each moment.
- *Consistency and Partition (CP)* Eventually the data stored on each partition must be consistent.
- *Availability and Partition (AP)* To ensure fault tolerance, the vertices and/or edges must be replicated on the nodes of the cluster.

On the other hand, for a complex network it becomes expensive to maintain the analysis requests on a single node because of the time latency and hardware requirement. To cope with this problem, the divide-and-conquer technique (divide-and-conquer) [19] can provide promising solutions. This technique consists in partitioning the graph into a set of sub-graphs and assigning them to the nodes of the cluster [18]. The first challenge of graph partitioning consists of finding a partition which minimizes the cut-edges between two subgraphs. It makes it possible to reduce the communication costs in the case of a high-performance computing [20]. The second challenge is the balancing of *k* partitions, it consists of having sub-graphs whose weights are closer. For $k=2$, it is a bi-partition of the graph. For large-scale graphs, this problem gets NP-complete [5, 6]. Once we seek to break the graph into $k\ge 3$ subgraphs, in polynomial time there will be no algorithm that can resolve the problem, and there is no exact solution. If we try to partition the graph into $k> 2$ subgraphs, there is no algorithm that can solve this problem in polynomial time and there is no exact solution [5, 6].

**Fig. 2:**
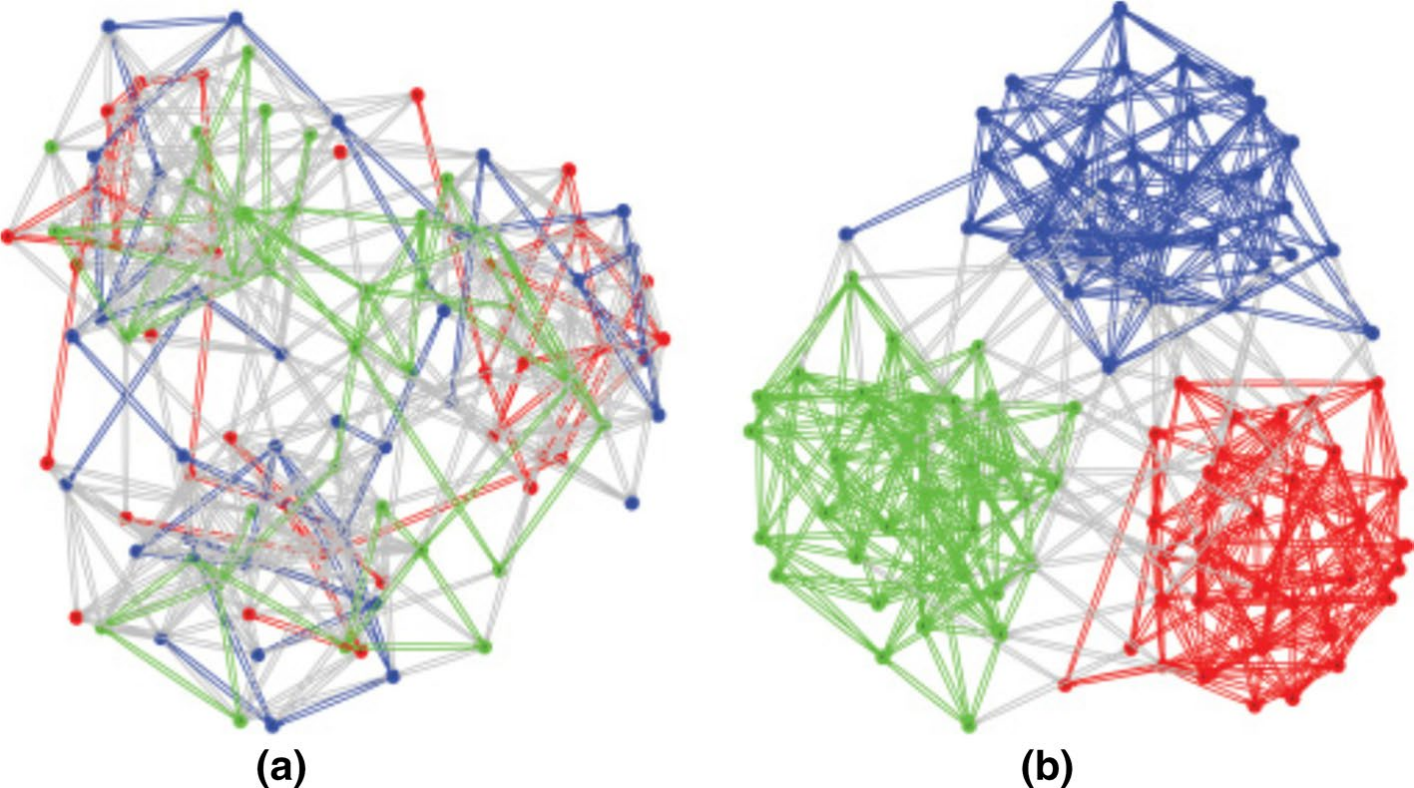
Illustration of an example of partitioning : **a** the partitioning is shoddy, the vertices are assigned randomly, there are more cut-edges between partitions; **b** the partitioning is almost optimal, the highly connected vertices reside in the same partition, there is less cut-edges [22]

Partitioning means either partitioning edges or vertices [8, 21]. In general, a k-partition corresponds to vertices partitioning [8]. Figure 2 illustrates examples of a 3-partition. The colors of vertices allow to identify the classes of the partition to which they belong. A distinct color is used for each distinct class. Colored edges represent the edges connecting pairs of vertices belonging to the same subgraph and gray edges represent the cut-edges between partitions [22]. We note respectively that the partitioning in Fig. 2a is poor compared to that of Fig. 2b. Note that each node stores a given partition, the cut-edges will be used for communication between nodes. Thus, a partitioning technique which minimizes the cut-edges and keeps the weight of the partitions almost equitably will thus allow to reduce the communication costs and will promote the load balancing between nodes [18].

### Contributions

We present a comparative analysis of existing methods for graph partitioning. Then, we present DPHV (Distributed Placement of Hub-Vertices) a distributed algorithm for large-scale graph partitioning which meets requirements load balancing and network bandwidth of the cluster nodes [4]. The experimental results performed on a multi-nodes cluster on real-world graphs show that our methodology is efficient and presents conclusive results compared to current distributed graphs partitioning algorithms such as Greedy [23], DFED [21] and JA-BE-JA [24].

### Organization

The rest of this article is arranged as follows. We provide some required background knowledge and explain the problem formulation in "Model and formalism" section. Furthermore, in "Related works" section, we address current methods of graph partitioning and provide a comparative analysis. Then in "Methodology" section, we introduce our graph partitioning methodology. In "Results and discussions" section, we evaluate the behavior and the performances of our method by experimental achievements. Finally, we conclude this paper with open challenges and future directions in "Discussions" section.

## Model and formalism

In this section, we provide some basic notions related to the graph partitioning problem. Then, we present a formalism of the k-partition problem.

### Definitions and notations

#### Definition 1

**(Graph)** A graph $Gr=(Vr, Ed)$ is a structure made of a set of vertices *Vr* and a set of edges $Ed = \{(v_1, v_2) | v_1, v_2 \in Vr \}$, which connects pairs of vertices from *Vr*.

Let |*Vr*| denote the number of vertices and |*Ed*| the number of edges of the graph.

In some situations, it may be useful to assign a weight *w* to each edge of the graph. A “*weighted graph*” is a graph $Gr = (Vr, Ed, W)$ with a weighting function $W:Ed{\mathop {\rightarrow }\limits ^{}}{\mathbb {R}}$ associated with the set of edges.

**Fig. 3:**
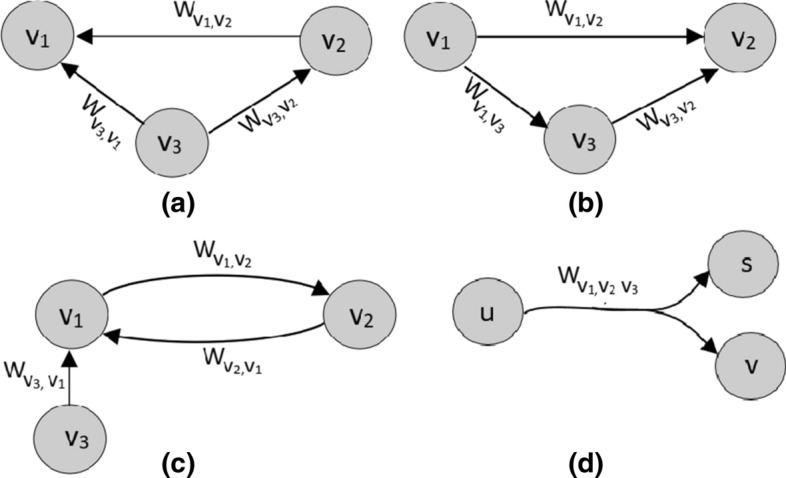
Various types of graphs: **a** undirected graph; **b** directed graph; **c** multi-graph; **d** hyper-graph

It is worth noting that the graphs may have various topologies regarding the edge characteristics. Figure  3 proposes a comparison of different types of weighted graphs. First, we may distinguish directed and undirected graphs. A graph is said to be undirected if the edge $(v_1,v_2)$ from vertex $v_1$ to $v_2$ corresponds to the edge from $v_2$ to $v_1$. If a pair of vertices are connected by more than two edges then the graph is said to be a “*multigraph*”. This form of graphs is more suitable for complex networks and is commonly used in NoSQL databases. An other special case of graphs is *“hypergraphs”*, which are graphs with hyper edges connecting more than two vertices at the same time [8].

#### Definition 2

**(Sub-graph)** Let $Gr=(Vr, Ed)$ be a graph, $Gr'=(Vr', Ed')$ is a sub-graph of *Gr* if and only if $Vr'$ is a subset of *Vr* and $Ed'$ is a subset of *Ed*. In other words, we obtain $Gr'$ by removing one or more vertices of *Gr*, as well as all the edges incident to these vertices.

**Fig. 4:**
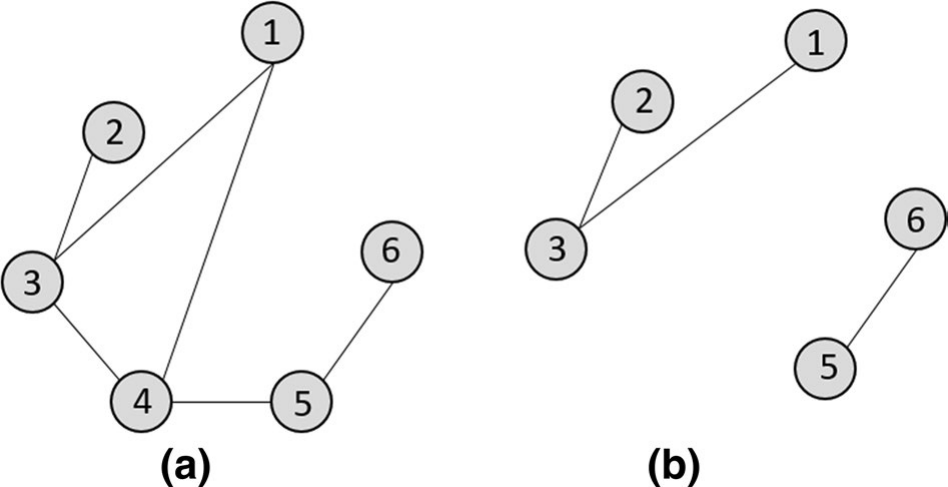
**a** Graph $Gr=(Vr,Ed)$; **b** sub-graph $Gr'=(Vr', Ed')$

In Fig. 4b, the graph $Gr'=(Vr', Ed')$ is a sub-graph of $Gr=(Vr, Ed)$ because $Vr' \subset Vr$ and $Ed' \subset Ed$. We obtain $Gr'$ by removing from *Gr* the vertex 4 and these adjacent edges (1, 4), (3, 4) et (4, 5).

#### Definition 3

**(Partition)** Let *Gr* be a nonempty set and *k* is a natural integer greater than or equal to 2. We say that $P_{k} = \{Gr_{1}, Gr_{2}, \ldots, Gr_{k}\}$ is a k-partition of *Gr* if:

- $$\forall i\in \llbracket 1;k \rrbracket$$, $$Gr_{i}\ne \emptyset$$
- $\forall i,j \in \llbracket 1;k \rrbracket$ such as $i\ne j$, we have $Gr_{i}\cap Gr_{j}$=$\emptyset$
- $$\bigcup \limits _{i=1}^{k} Gr_{i}= Gr$$

That is, the elements of $P_{k}$ are non-empty and pairwise disjoint.

### Formalism

Consider a data set given in the form of a big graph $Gr=(Vr, Ed)$ whose information is stored in *Vr* and *Ed* such that $n=|Vr|$ and $m=|Ed|$.

A partition $P_{k}=\{Gr'_{1},Gr'_{2} \ldots ,Gr'_{k}\}$ of the graph $Gr=(Vr, Ed)$ must highlight two fundamental properties:

- Balancing of sub-graphs $$Gr'_{i}$$$$\forall i \in \llbracket 1;k \rrbracket$$.
- Minimization of cuts $cut(Gr'_{i},Gr'_{j})$ between two sub-graphs $Gr'_{i}$ and $Gr'_{j}$.

Let $w(Gr'_{i})=|Ed'_{i}|$ be the weight of the $i^{th}$ sub-graph of *Gr*, of average weight $w_{avg}$ such that :

1 $$\begin{aligned} w_{avg} = \frac{\sum _{i=1}^{k} w(Gr'_{i})}{k} \end{aligned}$$

The load balancing $B(P_{k})$ of *k* partitions consists of calculating a k-partition $P_{k}=\{Gr'_{1},Gr'_{2}, \ldots,Gr'_{k}\}$ of *Gr* such as the weight of each sub-graph $Gr'_{i}$, $\forall i \in \llbracket 1;k \rrbracket$ contains at most $(1+\epsilon ).\frac{n}{k}$ vertices.

A partition $P_{k}$ is balanced if the constraint $B(P_{k})\le (1+ \epsilon )$ holds, that means the size of the sub-graphs are proportionally uniform with respect to a deviation error $\epsilon$.

A better way to measure if the weights of the sub-graphs are uniformly balanced is to use the standard deviation. It is a metric that measures the dispersion of the weights of the sub-graphs. It is defined as the quadratic mean of the deviations from the mean partition. It is calculated as follows :

2 $$\begin{aligned} B(P_{k}) = \sqrt{\frac{\sum _{i=1}^{k} \left( \frac{w(Gr'_{i})}{|Ed|/k} -1\right) ^2}{k}} \end{aligned}$$

**Lemma (Dispersion of partition weights)** Given a *k*-partition of a graph, the constraint of load balancing $B(P_{k})$ is not respected if for all sub-graphs $Gr'_{1}, \ldots, Gr'_{k}, \ldots Gr'_{k}$ of respective weight $w(Gr'_{1}), \ldots,w(Gr'_{k}), \ldots w(Gr'_{k})$, there is a sub-graph $Gr'_{i}$ whose weight causes the imbalance of $B(P_{k})$ such that $\forall i, j\in \llbracket 1;k \rrbracket$, we have:

3 $$\begin{aligned} w(Gr'_{i}) > |Ed|(\frac{\sqrt{k}}{k}(1+\epsilon ) +1) - \sum _{j\ne i}^{k}w(Gr'_{j}) \end{aligned}$$

#### Proof

In the case of a dispersion of the partition weights we have $B(P_{k})> 1+ \epsilon$

The second constraint is that of cut-edges, it consists of computing a partition $P_{k}$ which allows to minimize the cut-edges between two partitions $Gr'_{i}$ and $Gr'_{j}$. Moreover, it allows to reduce the communication costs in the case of a high-performance computation. It is calculated as follows:

4 $$\begin{aligned} cut(Gr'_{i},Gr'_{j}) = \sum \limits _{s_{i}\in Gr'_{i},s_{j}\in Gr'_{j}}(s_{i},s_{j}) \end{aligned}$$

where $(s_{i}, s_{j})$ corresponds to the cut between the sub-graphs $Gr'_{i}$ and $Gr'_{j}$. In this case, the overall cost of cut-edges the *k* sub-graphs is calculated as follows:

5 $$\begin{aligned} cut(P_{k}) = \sum \limits _{i,j\le k}cut(Gr'_{i},Gr'_{j}) \end{aligned}$$

The mathematical model of the k-partition problem with constraints could be encoded using the following system [8]:

6 $$\begin{aligned} {\left\{ \begin{array}{ll} \begin{aligned} &{} \text {min} &{} &{} cut(P_{k}) \\ &{} \text {subjects to:} &{} &{} B(P_{k})\le (1+\epsilon ) \\ &{} &{} &{} Gr'_{i}\cap Gr'_{j}=\emptyset \\ &{} &{} &{} \bigcup \limits _{i=1}^{k}Gr'_{i}=P_{k} \\ &{} &{} &{} Gr'_{i}, Gr'_{j}\ne \emptyset \\ &{} &{} &{} i\ne j \in \llbracket 1;k \rrbracket \end{aligned} \end{array}\right. } \end{aligned}$$

$$\square$$

## Related works

For some time now, the graph partitioning problem has aroused more interest because of NP-completeness [5] of the problem. Thus, numerous algorithms appeared [3]. In a survey paper, Adoni et al. [8] presented two search techniques: “local” or “global”. Local search algorithms begin with an arbitrarily chosen preliminary partition to progress towards a global graph partitioning (“vertex-centric” and “edge-centric”) [21]. The downside of this strategy is that the initial choice influences the quality of the obtained results [8]. In comparison, the global search approaches are based on the entire graph (“partition-centric” [21]).

**Fig. 5:**
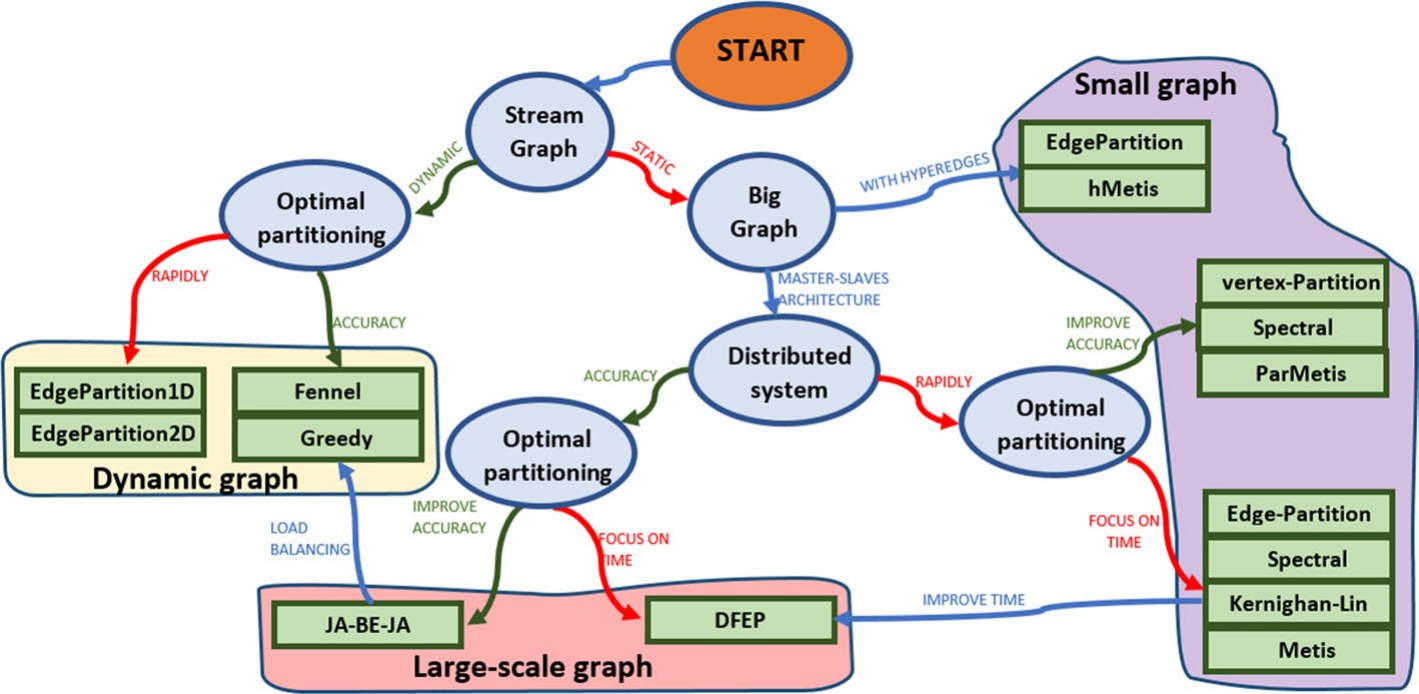
Roadmap of graph partitioning algorithms

The performance of graph partitioning algorithms is based on the time complexity or the result quality [5]. There are extremely fast algorithms whose solution is not optimal and slow algorithms which provide solutions close to the optimal. Adoni et al. [8] classified graph analysis algorithms into different categories as shown in Fig. 5.

The first category concerns classical methods, there are three mains: “vertex-partition”, “edge-partition” and “hypergraph-partition” [8, 21]. Vertex-partition consists of partitioning the set of vertices *Vr* [5]. The cut-edges between subgraphs are used as communication channels between cluster nodes. On the other hand, edge-partition partitions the set *Ed* of edges of the graph. So, the frontier vertices are used for information exchange across the cluster [21]. Vertex-partition method allows to have balanced partition $B(P_{k})$ while edge-partition minimizes the cut-edges. An extended version of edge-centric called hypergraph-partition concerns the partitioning of hypergraphs [21]. This algorithm works as edge-partition but used the hyper-edges as cut-edges [8].

The second one concerns the spectral clustering methods [25, 26]. Given a set of points $\{x_1, x_2, \ldots, x_n\}\in {\mathbb {R}}^n$, we consider an “affinity graph” $Gr=(Vr,Ed)$ such as the vertex $s_i \in Vr$ corresponds to the points $x_i \in {\mathbb {R}}^n$. The set of edges denotes the affinities between points and the weights related to each edge $(s_i, s_j)\in Ed$ encodes similarity values between $x_i$ and $x_j$. Spectral algorithm consists of steps. In the first step, we compute an affinity matrix *A*. Then, we compute the Laplacian matrix from *A*. Afterward, we extract the eigenvectors of *L*. Finally, we use these vectors for structural clustering.

Other algorithms [21, 27, 28] are based on partition via exchange. These algorithms are based on the optimization function. The choice of the initial solution influences the optimality of the result. Consequently, we may possibly fall into a local minima. Kernighan [27] used this concept to exchange the set of vertices of a given graph between k-partition while minimizing the cut-edges. Then we repeat the same task until, there are no exchanges that optimize the cut-edges function. Similarly, Fiduccia [28] presented an adapted version of “Kernighan algorithm” [27] for hypergraphs partitioning. Compared to Kernighan algorithm, it optimizes the hyper-edges function of the k-partition.

In another related works, the authors introduced [27, 29–32] multilevel partitioning methodology which may be adopted for partitioning the graph into subgraphs at each level. Karypis [30] follows this concept and proposed Metis. It is made of three steps: “coarsening” step, “partitioning” step and “refinement” step. Similarly, the authors proposed hMetis [31], an extended version of Metis [30] for multilevel partition of hypergraphs. Then, they introduced a parallel version of Metis that runs on multi-core processor [32].

Other graph partitioning algorithms are based on heuristic methods [8, 15, 33]. They are fast solving approaches but the result quality is not guaranteed to be optimal. As representative examples, we present EdgePartition1D and EdgePartition2D implemented in GraphX [15, 33]. These algorithms are fast and improved version of edge-partition which optimizes cut-edges using a hash function for edges partitioning. The partitioning strategy of this heuristic is the de-randomization of edge-partition which minimizes the cut-edges between subgraphs [8].

In addition to graph algorithms, some authors [23, 34, 35] introduced streaming algorithms [36] designed for partitioning dynamic graphs. Generally, dynamic graphs [36] are subject to frequent CRUD operations over the set of vertices and edges. Unfortunately, only a few number of methods [8, 23, 34, 35] are dedicated to dynamic graphs. Aggarwal et al. [35] proposed a clustering method for graph streams. They introduced a hash function based on the compression of new edges to improve the graph clustering. In the same way, Charalampos et al [34] proposed “Fennel”, a streaming graph partitioning algorithm. Fennel is founded on the optimization of an objective function which balances the weights of the subgraphs. Likewise, Joseph et al [23] presented a streaming algorithm based on power-law degree of distribution [8, 36]. The proposed strategy consists of giving priority to hub-vertices.

In the end, the last category concerns distributed partitioning algorithms [21, 22, 24, 37, 38]. Distributed algorithms are more effective for large-scale graphs because partitioning tasks are spread over cluster nodes. JA-BE-JA [22, 24] is a successful example of distributed algorithm and its implements a local search method that is based on simulated annealing [39]. It is fully decentralized, this allows the algorithm to be easily implemented into a distributed master-slaves architecture. The experimental results showed that JA-BE-JA is fast and the partition is balanced with less cut-edges. In spite of the performance of JA-BE-JA, it requires several hundred of iterations to converge towards an optimal partitioning. Therefore, it evolves costly communication overhead across the cluster nodes. To deal with this issue, Alessio [21] introduced DFEP, a distributed funding-edge partitioning algorithm. DFEP strategy consists of funding each subgraph by buying the edges of the graph at each iteration. DFEP requires less iterations to converge as compared to JA-BE-JA [24].

## Methodology

As presented in the previous section, there are several graph partitioning techniques. Some algorithms are fast but ineffective for use cases where the result optimality is more important than the time complexity. Likewise, there are very slow algorithms which provide almost optimal results. Until then, the partitioning techniques not yet studied are the parallel and distributed approaches [40]. For the moment, JA-BE-JA [22, 24] is the best big graph partitioning technique but its approach based on simulated annealing is much more iterative [39]. In some cases, it will be necessary to wait several hundred iterations in order to obtain a result within the limits of satisfaction constraints. Which can be very costly on time and consumes a lot of hardware resources.

In this section, we introduce DPHV (Distributed Placement of Hub-Vertices), a distributed and parallel heuristic suited for partitioning of large-scale graph according to vertex-centric paradigm and uses a monitoring agent which ensures that the weight constraints of each partitions is within normal limits. DPHV is scalable, designated for intensive computation. The partitions are strongly connected inside. In addition, it can also be implemented according to the partition-centric paradigm [40].

The proposed algorithm is parallel and distributed in a multi-nodes cluster. DPHV is based on the placement of hub-vertices. The objective of this approach is to propose partitioning according to the following criteria [3]:

- *Partition balancing* [41] the partition weights must be as close as possible. This makes it possible to have the same computational loads on each node.
- *Communication costs* [38, 42]: the exchange of information between two partitions is done through cut-edges. Each partition cut increases communication costs, which risks causing a network bottleneck in the event of a high number of cuts. Our goal is to minimize these cuts.
- *Connectivity* [37]: the sub-graphs induced in each partition must remain connected as much as possible as well as the clicks. This condition is not a necessity but it allows to preserve the topology of the original graph.

The balancing of the weights of the partitions can be done simply by a random placement of the vertices so as to have partitions of weight close to $\frac{|Ed|}{k}$ [8]. However, this will involve serious communication costs between partitions and not guarantee that the topology of the graph will be preserved [13]. The proposed approach takes these two compromises into account. Since the partitioning problem is considered as an NP-complete problem because of the fact that there is no exact resolution method in polynomial time. The applicability of this problem in the case of a large-scale graph is expensive and the computation time is considered impractical [5]. Generally, it takes several iterations to converge towards a quasi-optimal solution [21]. It is also important to emphasize that the choice of the initial solution can lead to a local optimal problem. For example, partitions that start near the center of the graph will tend to explore more space than partitions that start at the edges of the graph.

**Fig. 6:**
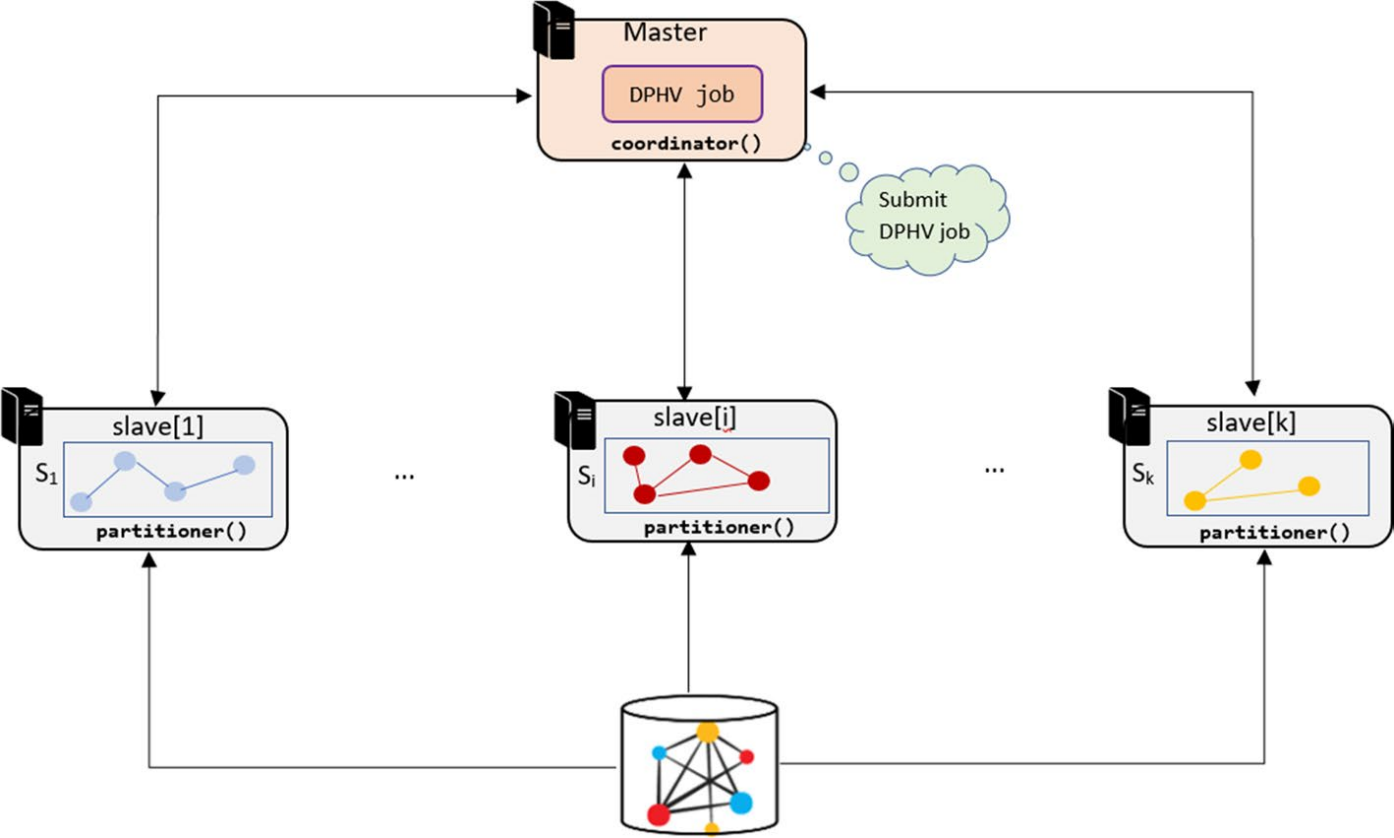
Overview of the DPHV execution framework

To face these challenges, we introduced DPHV, an algorithm based on the placement of hub vertices, that is to say the vertices which have a great impact on the weight and the topology of the graph [8]. DPHV is based on vertex-partition method and implemented according to vertex-centric paradigm [24]. It is an iterative algorithm which at each iteration places *k* vertices on *k* partitions. DPHV is completely decentralized, each slave node is responsible for placing the vertex which will cause fewer cut-edges, while the master node is responsible for coordinating and monitoring the partitioning so as to have partitions of almost similar weight [13]. The vertices are sorted according to the order of their degree in the pre-processing phase, this allows derandomization of the placement and avoids local optimum problems. The hub-vertices that is to say having a high degree are placed as a priority. This also allows to change the graph exploration strategy. Unlike other partitioning algorithms that explore from boundaries to the center of the graph, DPHV explores in the direction of the hub vertices towards the vertices less impacting on the topology of the original graph [13]. This allows in some cases to preserve the topology of the graph in a distributed way and the connectivity between the vertices residing in the same partition. DPHV is designed to run on master-slaves architectures, as illustrated in Fig. 6. DPHV algorithm is composed of two parts: coordinator() and partitioner().

The parameters used in the DPHV heuristic pseudo-code are :

- $G=(Vr, Ed)$: graph composed of set of vertex *Vr* and edge *Ed* such that $n =|Vr|$ and $m =|Ed|$.
- *k*: number of sub-graphs of the graph *Gr* such that $k>2$. It is a hyperparameter which impacts the execution time and the result optimality.
- $Gr'_{i}$: sub-graph *i*, such as $i\in \llbracket 1; k \rrbracket$ the weight of each partition is defined by $w(Gr'_{i})$.
- *slave*[*i*]: slave node which hosts the partition $Gr'_{i}$.
- $s_{i}$: vertex assigned to the partition $Gr'_{i}$, such as $s_{i} \in Vr$. We denote $Vr(s_{i})$ the set of these adjacent vertices such that $d(s_{i})$ is its degree.
- *M*: contains all the vertices that have been assigned to one of the *k* partitions.
- $Cut_{s_{i}}(Gr'_{i})$: number of cut-edges generated by the assignment of the vertex $s_{i}$ in the partition $Gr'_{i}$.
- $In_{s_{i}}(Gr'_{i})$: number of induced edges generated by the assignment of $s_{i}$ to the sub-graph $Gr'_{i}$.
- $f_{R}(Gr'_i, s_i)$: ratio function of the number of induced edges compared to the number of cut-edges generated by the assignment of $s_{i}$ to $Gr_{i}$. It is calculated as follows:
  7 $$\begin{aligned} f_{R}(Gr'_i,s_i)=\frac{In_{s_{i}}(Gr'_{i})}{Cut_{s_{i}}(Gr'_{i})} \end{aligned}$$
  such as $Cut_{s_{i}}(Gr'_{i}) = 1$ if there is no cut-edge generated by the assignment of the vertex $s_i$ to the sub-graph $Gr'_{i}$. Moreover, if no edge is generated inside the subgraph $Gr'_{i}$, then $In_{s_ {i}}(Gr'_{i}) = 1$.

### The load balancer

The coordinator() program is centralized on the master node, it is responsible of monitoring the state of the slave nodes and ensures that the weight of the partitions is equitably balanced across the cluster.

Algorithm 1 describes the process of coordinator(). Initially all nodes into the cluster are in the active state. So, at each iteration, the coordinator evaluates the value of the partition balance $B(P_{k})$. If the balance constraints of the partition with respect to the acceptance error $\epsilon$ are not respected (see section 2) then, the coordinator checks at each iteration whether the weight $w(Gr'_{i})$ of a subgraph $Gr'_{i}$ is not far from the average or is not too high compared to the other subgraphs. Then the coordinator puts it in inactive state via the haltNode(true, slave[i]) method.

When the subgraphs weights are balanced by comparison with the partition weight $Gr'_{i}$ then the coordinator puts the node *slave*[*i*] in active state. Once all the vertices of the graph have been placed or marked, the coordinator deactivates all the slave nodes of the cluster and signals the end of the partitioning job.

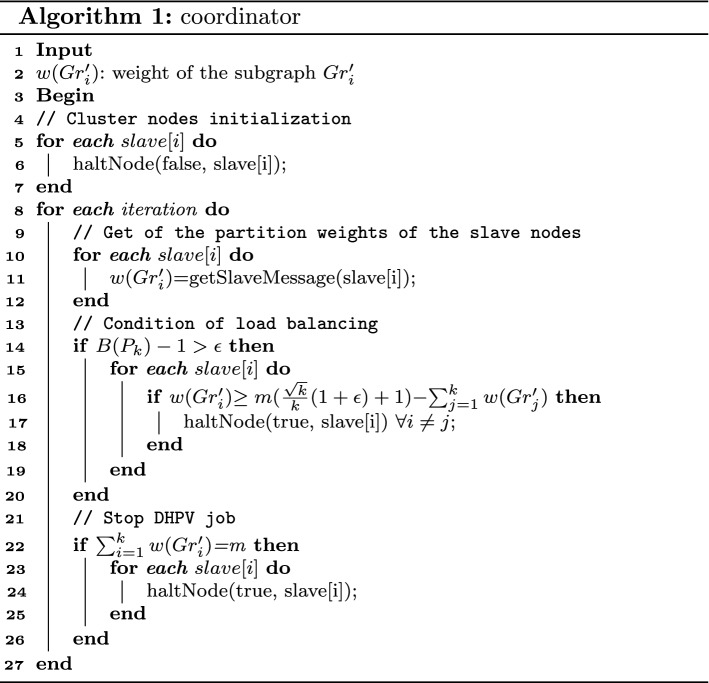



### The distributed partitioning strategy

Unlike the coordinator() which is centralized on the master node, partitioner() is decentralized on all the slave nodes of the cluster.

partitioner() processes and assigns each vertex before proceeding to the next one, it keeps in memory the current weight of its partition. In the event of q compromise, the following rules are used in the placement decision:

- If the majority of the neighbors of the current vertex are already in a subgraph $Gr'_{i}$, then the vertex will be added to this partition;
- If it has no subgraph in common, the subgraph with the most edges associated with this vertex will be chosen;
- If the vertex assignment generates the same placement ratio for all subgraphs, then the vertex will be assigned to the smallest subgraph $Gr'_{i}$ such that $w(Gr'_{i}) = min\{w(Gr'_{1}), w(Gr'_{2}), \ldots, w(Gr'_{k})\}$;
- Otherwise the vertex will be randomly assigned to one of the *k* subgraphs.

It is important to emphasize that the graph provided as input is supported by the distributed storage system of Hadoop HDFS but without physically partitioning the graph. In this case, the size of the block file plays an important role because it defines the size of the sub-blocks making up each piece of data stored on the nodes of the cluster. This allows each node to have a global view on each block of the original graph and to ensure better information exchange between nodes. The data format of the graph supplied to enter is based upon the “Extended Property Graph Model” (EPGM) [43] model. Long before the partitioning phase, the vertices of the graph are ordered in ascending order of the degree of each vertex. This is done via a quick sorting by insertion operation whose execution time complexity is *O*(*nlog*(*n*)) et $O(n^2)$ in the worst case.

Algorithm 2 presents the pseudo-code of partitioner() program. Initially, each partition $Gr'_{i}$ is empty as well as the associated weight $w(Gr'_{i})$ and the node *slave*[*i*] receives a message from the master node notifying the start of the partitioning task. At each iteration, as long as the node *slave*[*i*] does not receive a message signaling the end of the job, then for each *k* unmarked vertices, we evaluate the ratio $f_{R}(Gr'_i, s_i)$ of the number of induced edges generated by the placement of this vertex $s_{i}$ in the partition $Gr'_{i}$ compared to the number of cut-edges generated by the placement of the vertex $s_{i}$. Then the vertex $s^*_{i}$ having the maximum value of $f_{R}(Gr'_i,s^*_{i})$ is chosen. If two slave nodes *slave*[*i*] and *slave*[*j*] whose vertices $s^*_{i}$ and $s^*_{j}$ are promising and if $w(Gr'_{j}) \ge w(Gr'_{i})$ then the vertex $s^*_{i}$ will be placed in the partition $Gr'_{i}$ while the vertex $s^*_{j}$ will be replaced by the vertex $s_{j-1}$ and placed in the partition $Gr'_{j}$. Each of the *k* vertices placed is marked. Subsequently we add all incident edges to vertex $s_{i}$ as well as the cut-edges generated by the assignment of $s_{i}$. Finally, the slave node *slave*[*i*] communicates by message the new value of the weight of its partition to the master node. It is important to emphasize that the partition task is parallelized according to BSP (Bulk Synchronous Parallel) paradigm [41]. So when a node finishes placing a vertex, it waits until the rest of nodes finish their job. Thus, the time complexity of each node is $O(\frac{|Vr(s_{i})|n^2}{k}log(k))$.

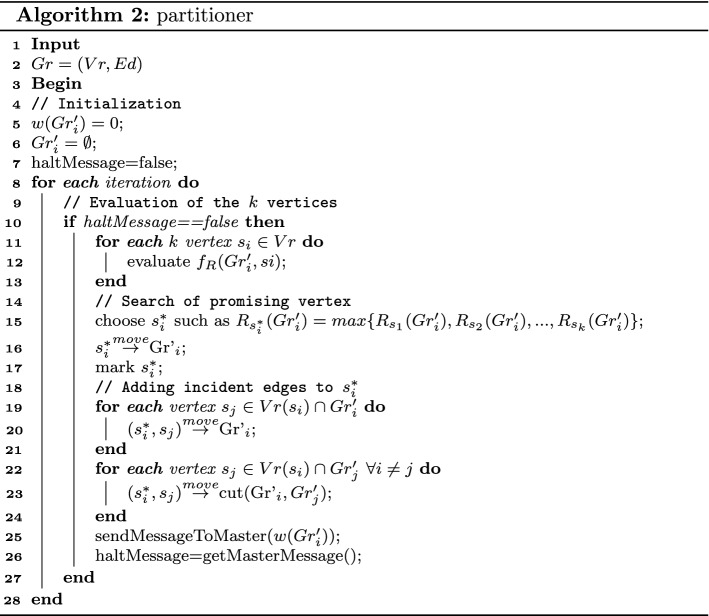



## Results and discussions

### Illustration of DPHV algorithm

**Fig. 7:**
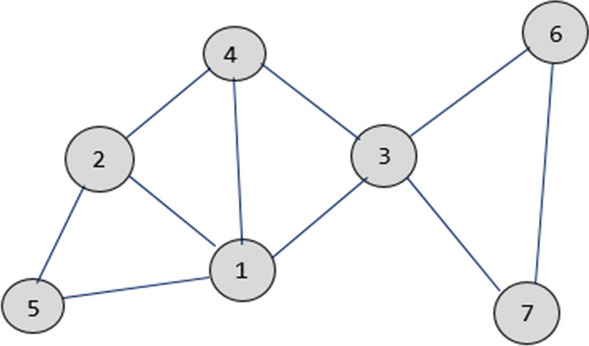
Graph $Gr=(Vr,Ed)$ such as $|Vr|=7$ et$|Ed|=10$

We highlight a simple illustration of DPHV algorithm for $k= 2$. Let $Gr =(Vr, Ed)$ be a graph composed of 7 vertices and 10 edges which we want to partition into 2 sets $Gr'_{1}$ and $Gr'_{2}$. It is assumed that the cluster used is set up of 2 slave nodes which perform the partitioning task while the master node supervises the DPHV job (Table 1, Fig. 7).

#### The pre-processing phase

This phase consists of ordering the 7 vertices of the graph in descending order of their respective degrees. Table 2 presents the vertices as well as the degree of each vertex. Initially the two partitions are empty and their respective weights are $w(Gr'_{1}) = w(Gr'_{2}) = 0.$

**Table 1:** Degree of vertices of the graph

$s_{i}$	1	3	2	4	7	5	6
$d(s_{i})$	5	4	3	3	3	2	2

#### The partitioning phase

**Fig. 8:**
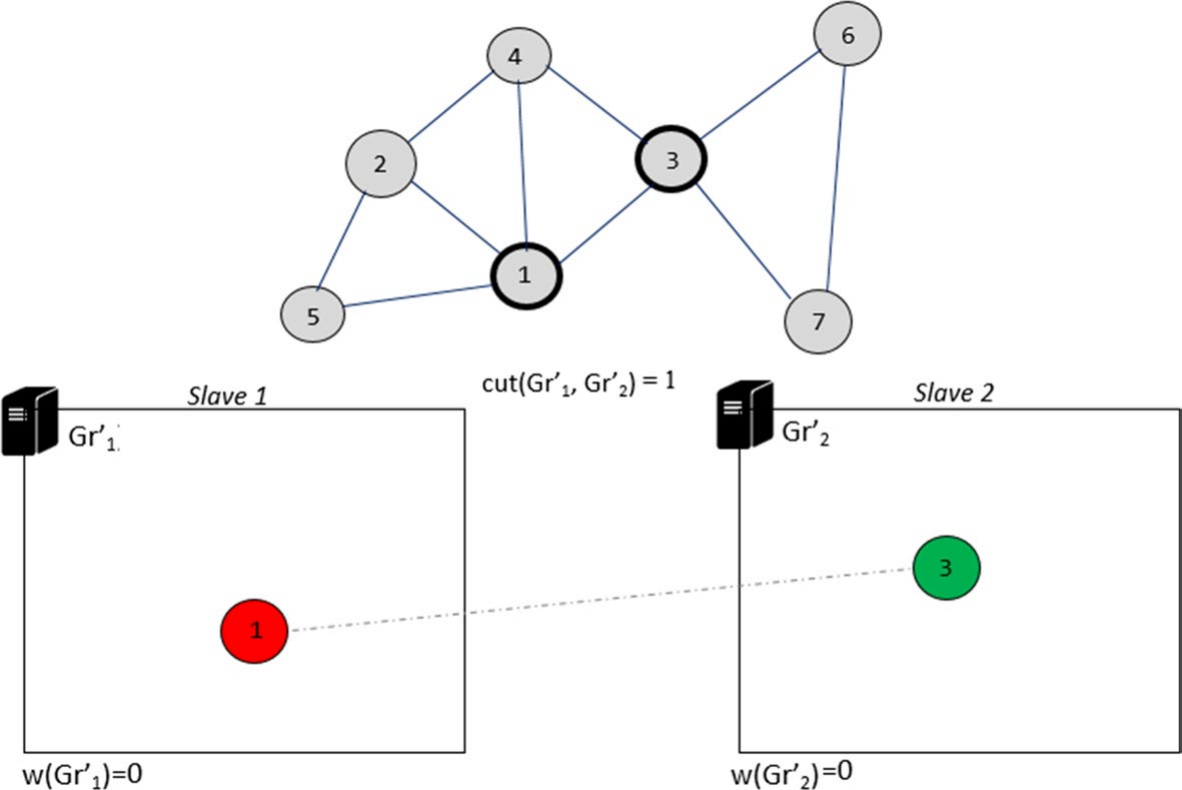
Iteration 1 of DPHV execution

Iteration 1: initially each slave node randomly selects the first $k = 2$ vertices $\{s_1, s_3\}$ not marked. In this example, vertex $s_1$ is assigned to the partition $Gr'_{1}$ while vertex $s_3$ is assigned to the partition $Gr'_{2}$. Then the internal edges and cut-edges are added. Once this task is completed, the two vertices will be marked (Fig. 8).

Iteration 2: then the other two unmarked vertices $\{s_2, s_4 \}$ are selected. Thus, in parallel the two slave nodes evaluate the following operations:

$$s_2 {\mathop {\rightarrow }\limits ^{move}} slave[1] \Longrightarrow f_{R}(Gr'_1,s_2)=1$$$$(Cut_{s_2}(Gr'_{1})=1, In_{s_2}(Gr'_{1})=1)$$

$$s_4 {\mathop {\rightarrow }\limits ^{move}} slave[1] \Longrightarrow f_{R}(Gr'_1,s_4)=1$$, $$(Cut_{s_4}(Gr'_{1})=1, In_{s_4}(Gr'_{1})=1)$$

$$s_2 {\mathop {\rightarrow }\limits ^{move}} slave[2] \Longrightarrow f_{R}(Gr'_2,s_2)=1$$, $$(Cut_{s_2}(Gr'_{2})=1, In_{s_2}(Gr'_{2})=1)$$

$$s_4 {\mathop {\rightarrow }\limits ^{move}} slave[2] \Longrightarrow f_{R}(Gr'_2,s_4)=1$$$$(Cut_{s_4}(Gr'_{2})=1, In_{s_4}(Gr'_{2})=1)$$

**Fig. 9:**
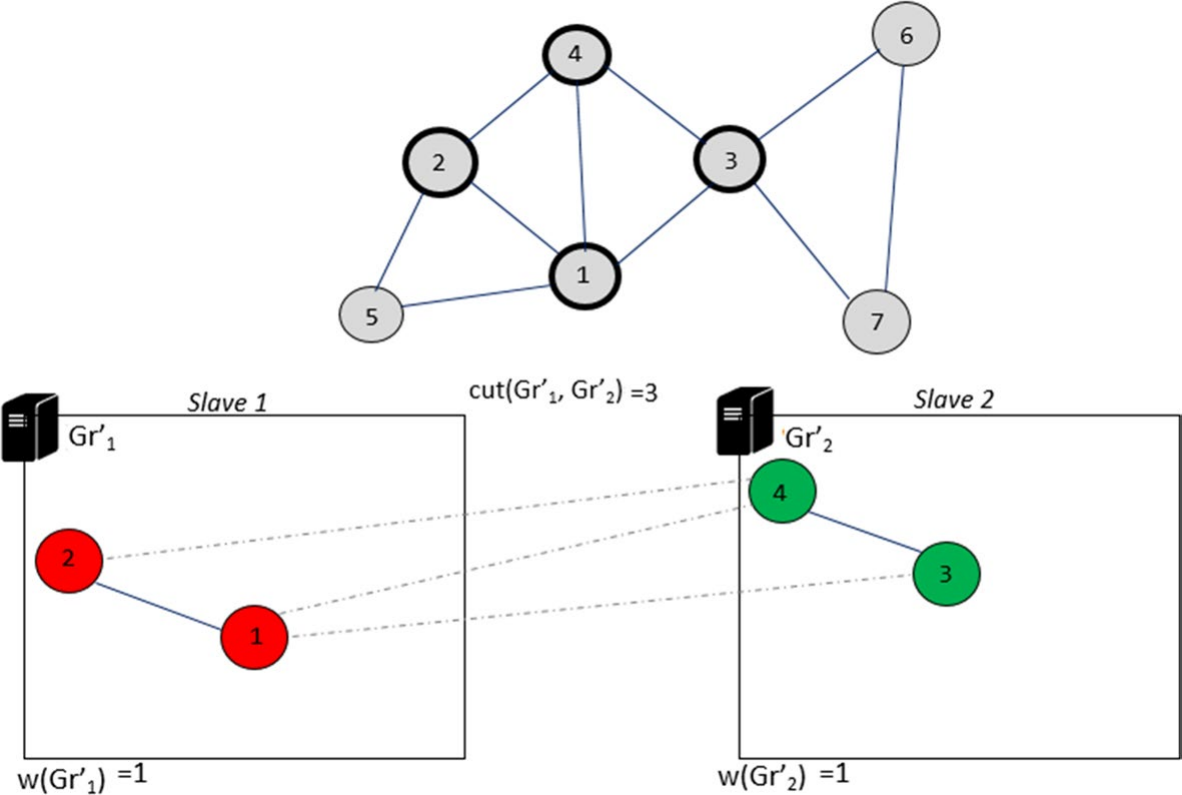
Iteration 2 of DPHV execution

The vertices $s_2$ and $s_4$ are marked and placed respectively in the partitions $Gr'_{1}$ and $Gr'_{2}$ (Fig. 9).

Iteration 3: We repeat the same operations again by selecting the $k = 2$ unmarked vertices $\{s_5, s_7\}$. Each slave node then evaluates the best placement:

$$s_7 {\mathop {\rightarrow }\limits ^{move}} slave[1] \Longrightarrow f_{R}(Gr'_1,s_7)=1$$$$(Cut_{s_7}(Gr'_{1})=1, In_{s_7}(Gr'_{1})=1)$$

$$s_5 {\mathop {\rightarrow }\limits ^{move}} slave[1] \Longrightarrow f_{R}(Gr'_1,s_5)=2$$$$(Cut_{s_5}(Gr'_{1})=1, In_{s_5}(Gr'_{1})=2)$$

$$s_7 {\mathop {\rightarrow }\limits ^{move}} slave[2] \Longrightarrow f_{R}(Gr'_2,s_7)=1$$$$(Cut_{s_7}(Gr'_{2})=1, In_{s_7}(Gr'_{2})=1)$$

$$s_5 {\mathop {\rightarrow }\limits ^{move}} slave[2] \Longrightarrow f_{R}(Gr'_2,s_5)=1$$$$(Cut_{s_5}(Gr'_{2})=1, In_{s_5}(Gr'_{2})=1)$$

**Fig. 10:**
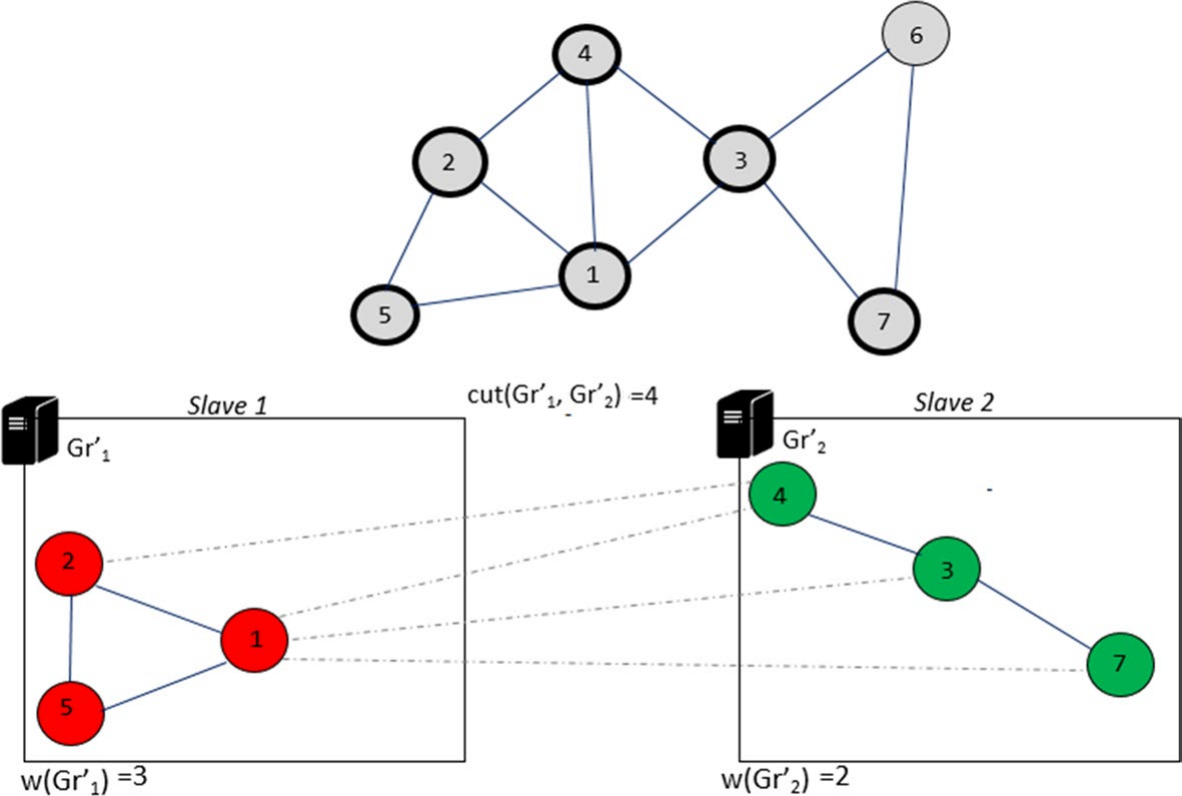
Iteration 3 of DPHV execution

The vertices $s_5$ and $s_7$ are marked and placed respectively in the partitions $Gr'_{1}$ and $Gr'_{2}$ (Fig. 10).

Iteration 4: Finally, in the last part, the last unmarked vertex $s_6$ is evaluated in parallel to determine the placement which generates less cut-edges:

$$s_6 {\mathop {\rightarrow }\limits ^{move}} slave[1] \Longrightarrow f_{R}(Gr'_1,s_6)=1$$$$(Cut_{6}(Gr'_{1})=2, In_{s_6}(Gr'_{1})=0)$$

$$s_6 {\mathop {\rightarrow }\limits ^{move}} slave[2] \Longrightarrow f_{R}(Gr'_2,s_6)=2$$$$(Cut_{s_6}(Gr'_{2})=1, In_{s_6}(Gr'_{2})=2)$$

**Fig. 11:**
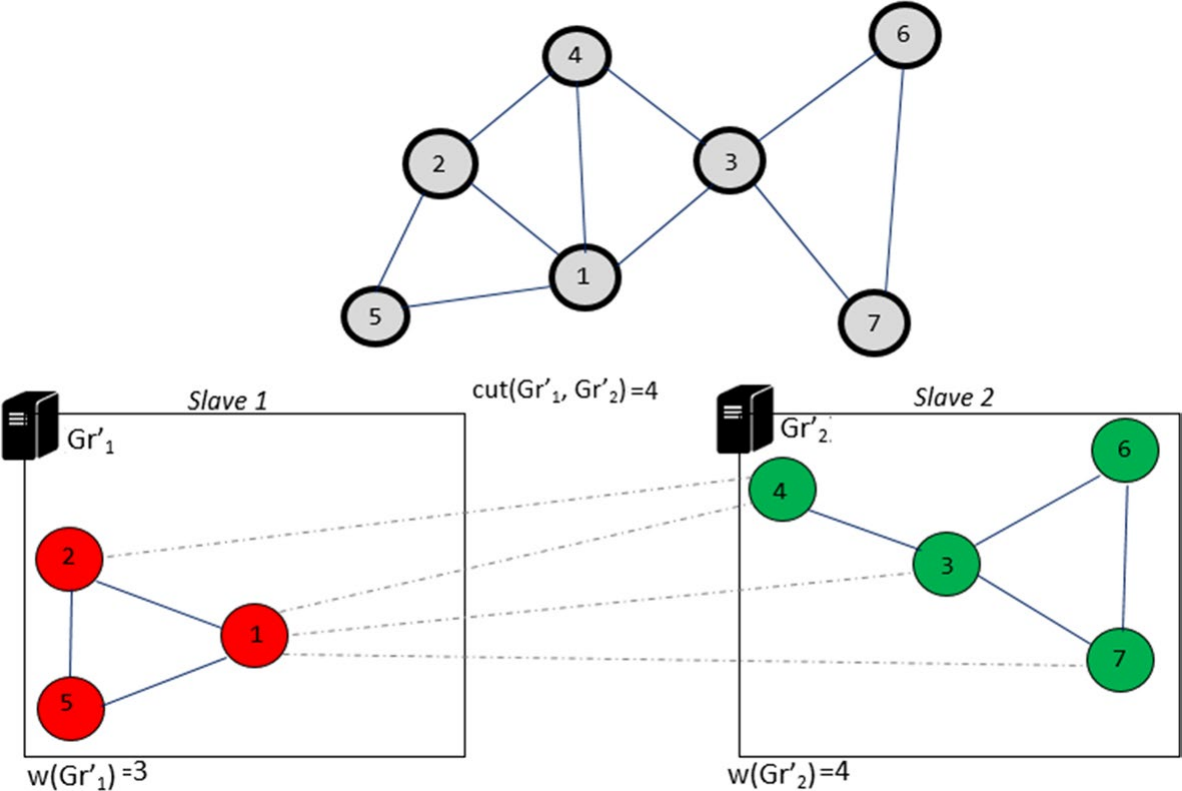
Iteration 4 of DPHV execution

The vertex $s_6$ is placed in the partition $Gr'_{2}$ because it generates less cut-edges. The algorithm stops because all vertices are marked. The solution obtained is one of the solutions that DPHV can generate. Another solution would be to place the vertex $s_4$ in the partition $Gr'_{1}$, this will reduce the number of cut-edges to $w(Gr'_{1}, Gr'_{2}) = 3$ (Fig. 11).

The main drawback of DPHV is that it does not optimize the solution obtained in order to get as close as possible to the optimal. On the other hand, it allows the placement of a dynamic graph according to the same paradigm. DPHV is even faster when the number of partitions is small. When the number of partitions increases, the master node performs more operations because it will be necessary to regulate the weight of each partition so as to remain within the limits of acceptance constraints. When for example *l* slave nodes go into inactive state, the problem of *k*-partition automatically switches to a problem of $k'$-partition with $k'=k-l$. In addition, unlike other partitioning strategies which perform random placement of vertices or edges, DPHV is completely de-randomized, which means that regardless of the topology of the graph, the algorithm cannot fall into a local optimal.

### Test environment and dataset

**Fig. 12:**
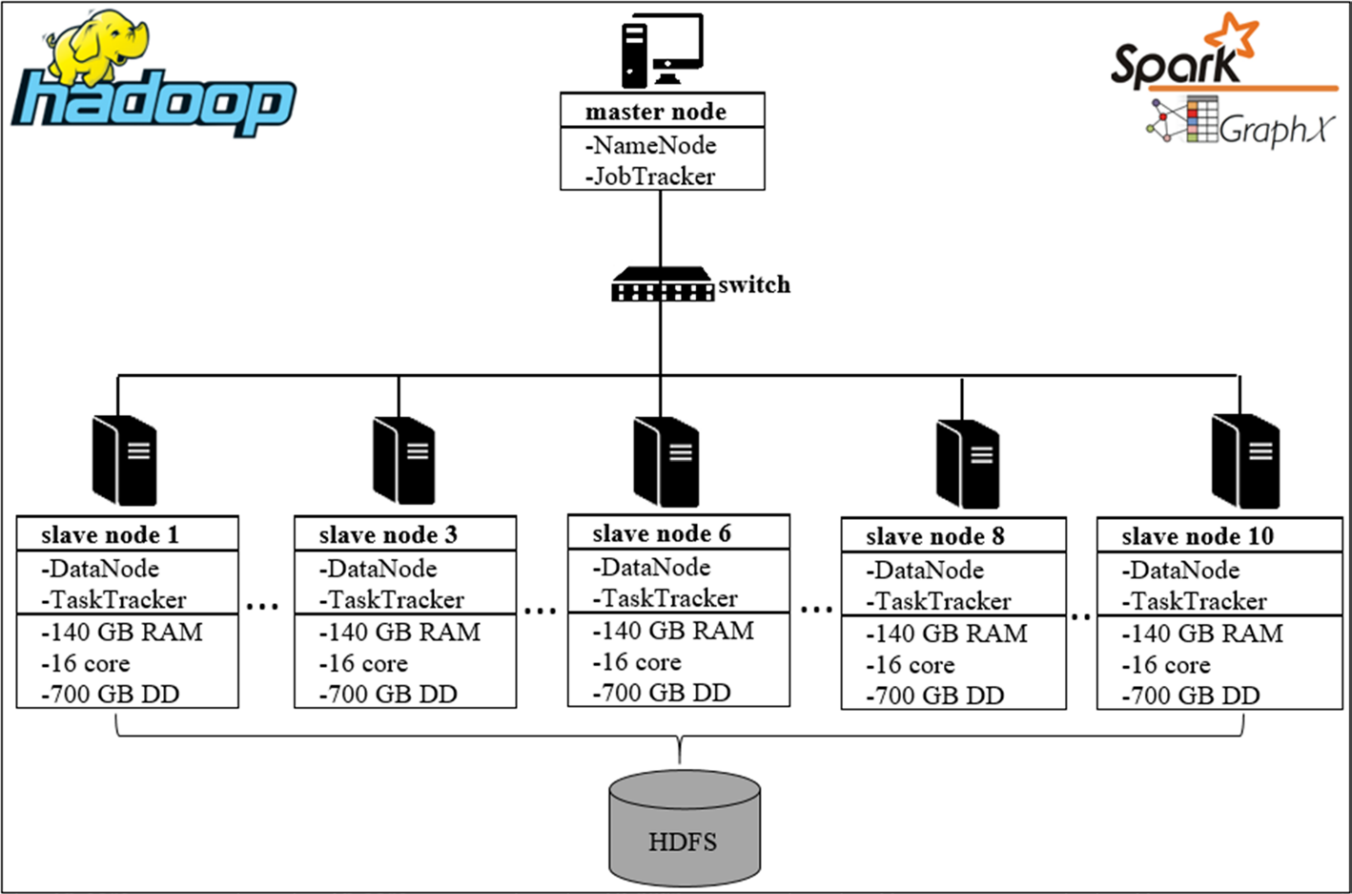
Test environment [14, 15]

The experimental tests were carried out on the Grid’5000^1^ an open platform for cloud computing. It is dedicated exclusively to experimental tests involving high performance computations on parallel and distributed systems. This platform already incorporates big data platforms like Spark [15], GraphX [33] and Hadoop [14]. The cluster allocated for the tests contains 10 nodes configured in a homogeneous manner (see Fig. 12). The experimental tests were also carried out by changing the number of nodes. Each node is equipped with 240 GB SSD + 480 GB SSD + 4.0 TB HDD, 140 GB of RAM, 10 Gbps + 100 Gbps of Omni-Path Ethernet cables and a 2 x Intel Xeon Gold 6130 (16 cores/ CPU). We adopted Ganglia [42] for monitoring the cluster’s performance.

We used benchmark data extracted from SNAP^2^ a large network dataset collection. To highlight the applicability and the performances of our heuristic, we used two categories of datasets. The first category represents deterministic finite automata with explosion of states. These data were extracted from the set of different conformance test models of various complex systems^3^. These datasets correspond to the finite behavioral models of the test tools piloted by formal verification models for the performance of conventional test tasks: selection of test cases, prioritization, mutation tests, etc. The second category of datasets used represents the road network of Morocco collected from the OpenStreetMap (OSM) spatial database^4^. It contains points, different types of roads and lanes between two points of interest. Each entity of the road network contains tags nested in each of these objects. The graph of the road network covers all types of road, including local roads. It contains directed and weighted edges to estimate distances/time of travel.

Table 2 presents the characteristics of the graphs extracted from the datasets. For each dataset, we present the number of vertices |*Vr*|, the number of edges |*Ed*|, the diameter of the graph *D* and the clustering coefficient *ACC*.

**Table 2:** Datasets

Designation	Type	$\mathbf |Vr|$	$\mathbf |Ed|$	ACC	D
Biosnap	Undirected	1018524	24735503	0.42	15
Twitter	Directed	81306	1768149	0.5653	7
Usroad	Directed	126146	161950	0.0145	617
Email	Undirected	36692	183831	0.4970	11
Astro	Undirected	18772	198110	0.6306	14
ageRRN	Directed	860000	2360000	56	0.4970
cptTRM	Directed	1070000	6000000	70	0.6235
elsaRR	Directed	1508000	9000000	92	0.5653
osmMA	Directed	4526700	12670000	412	0.0136

### Complexity of graph partitioning algorithms

**Fig. 13:**
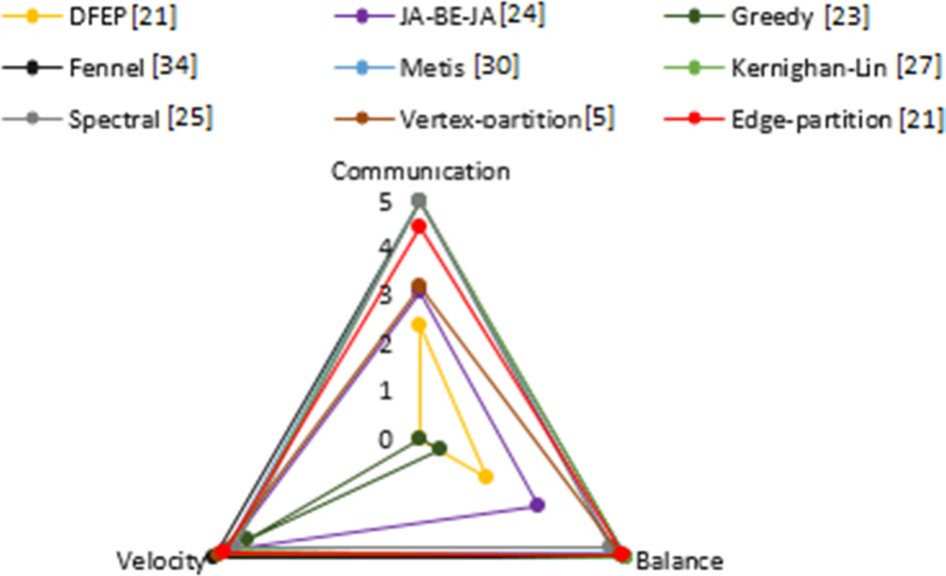
Computational complexity of graph partitioning methods

Vertex-partition [5], Edge-partition [21], Spectral [25], Kernighan-Lin [27], Metis [30] and Greedy [23] methods were implemented in python 3.4. DFED [21] method was implemented in Java 8.2 for MapReduce [14] version and Scala 2.12 for Spark in-memory version [44]. JA-BE-JA [24] method was also written in Scala 2.12. Each network is partitioned under $k = 25$ partitions. The algorithms have been executed ten times to get an average parameter values: the runtime, the cost communication and the load balancing. A partitioning method is considered effective if it is both fast and results in balanced partitions with fewer cut-edges. Figure 13 illustrates the performance of different techniques according to the hyper parameters. The methods that can satisfy these three constraints are: Vertex-partition [5], Edge-partition [21], Spectral [25], Metis [30], Kernighan-Lin [27] and Fennel [34]. The performances of JA-BE-JA [24], DFED [21], Greedy [23] vary according to the graph topology. For example, Greedy [23] is very fast in terms of velocity but generates a large number of cut-edges and the partition is not balanced.

**Fig. 14:**
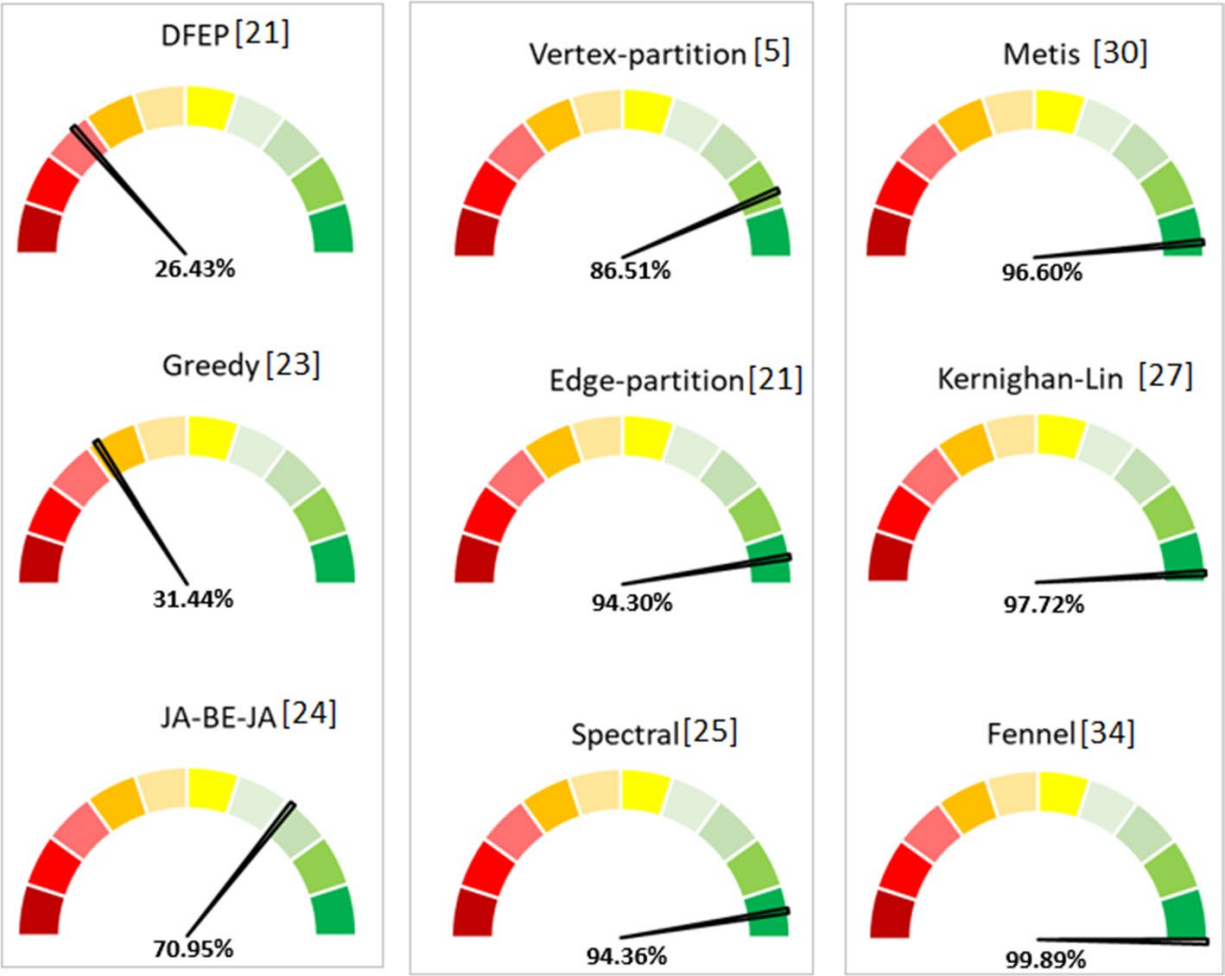
Constraints satisfaction rate

Figure 14 introduces for the constraints satisfaction rate of each graph partitioning method. The performance of each algorithm is the result of crossing the three evaluation parameters. The rate of the maximum performance obtained varies from 1 to 100%. A rate close to 100% means that all of the constraints are satisfied.

We observe that Fennel [34], Metis [30], Kernighan-Lin [27] and Spectral [25] have a satisfaction rate that ranges between 94% and approximately 100%. This highlights the previous results analysis. DFED [21] and Greedy [23] have a lower constraints satisfaction rate. However, it is important to emphasize that this rate can vary depending on the graph topology and the cluster configuration. A performance rate below the average does not necessarily imply that the method is ineffective. There are a number of trade offs in choosing an appropriate graph partitioning method [8]. This involves in-depth reflection in the pre-processing phase.

### Computational complexity of DPHV

The study of the complexity of DPHV was carried out on osmMA dataset in order to better understand its complexity of our algorithm. We opted for this dataset because it is a complex network which puts all the evaluation parameters of our approach into competition. We also analyze the behavior of DPHV with respect to the variation in the number of partitions *k*. Then we make a comparison with benchmark models of existing distributed partitioning algorithms. The evaluation parameters highlighted are 1) the time complexity; 2) communication costs of the cluster, 3) the load balance and 4) the connectivity of the sub-graphs induced in each partition [5, 6]. DPHV program was written in Java and the different Jobs run on JVM 1.8.

**Fig. 15:**
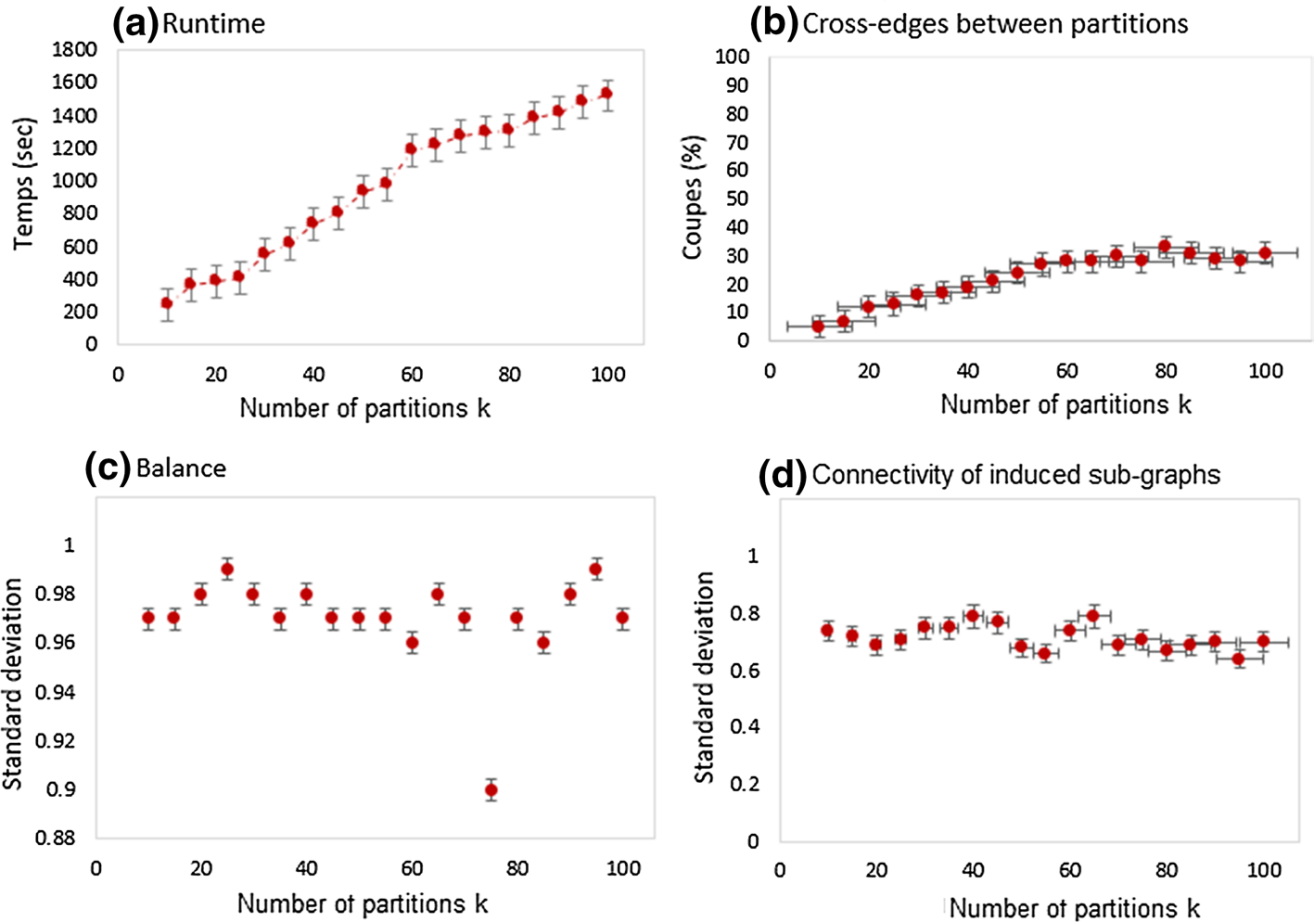
DPHV behavior based on the number of partition *k*

Figure 15 shows the behavior of DPHV compared to the number of partitions. Note that the variation of the number of partitions has a great impact on the behavior of DPHV. When the number of partitions increases, the algorithm tends to run slowly, which is quite logical since the time taken to partition a graph into $(k + 1)$ partitions is significant than that of a *k* partitions. In addition, for a high *k*, DPHV will spend more time evaluating a large number of vertices at each iteration. This greatly influences the time complexity but allows obtaining a better partition which minimizes cut-edges. We also note that whatever the variation of the number of partitions, the percentage of cut-edges varies between 20% and 30%. This is remarkable because it demonstrates that DPHV manages to stabilize the evolution of cut-edges, thus making it possible to reduce or control the costs of communication.

The standard deviation in Figure 15c oscillates between 0.9 and 0.98, this value is very close to 1 whatever the number of partitions. We can conclude that DPHV balances the partition weights so that the constraint $B(P_{k})\le (1+ \epsilon )$ is respected. This aspect is very important because it ensures good parallelism [8, 40] with workloads distributed evenly on each node of the cluster. This reduces the latency the time spent in synchronization tasks.

Figure 15d shows that the standard deviation oscillates between 0.6 and 0.8, this means that the connectivity of each subgraphs induced in each partition remains close from that of the original graph. In addition, our heuristic is the first to propose partitions whose vertices induced in each partition are strongly connected.

DPHV is able to partition large-scale graphs in a parallel and distributed architecture, all while preserving the graph topology as much as possible. While optimizing the number of cut-edges in order to minimize the communication costs. In addition, DPHV is scalable and supports the large-scale graph.

**Fig. 16:**
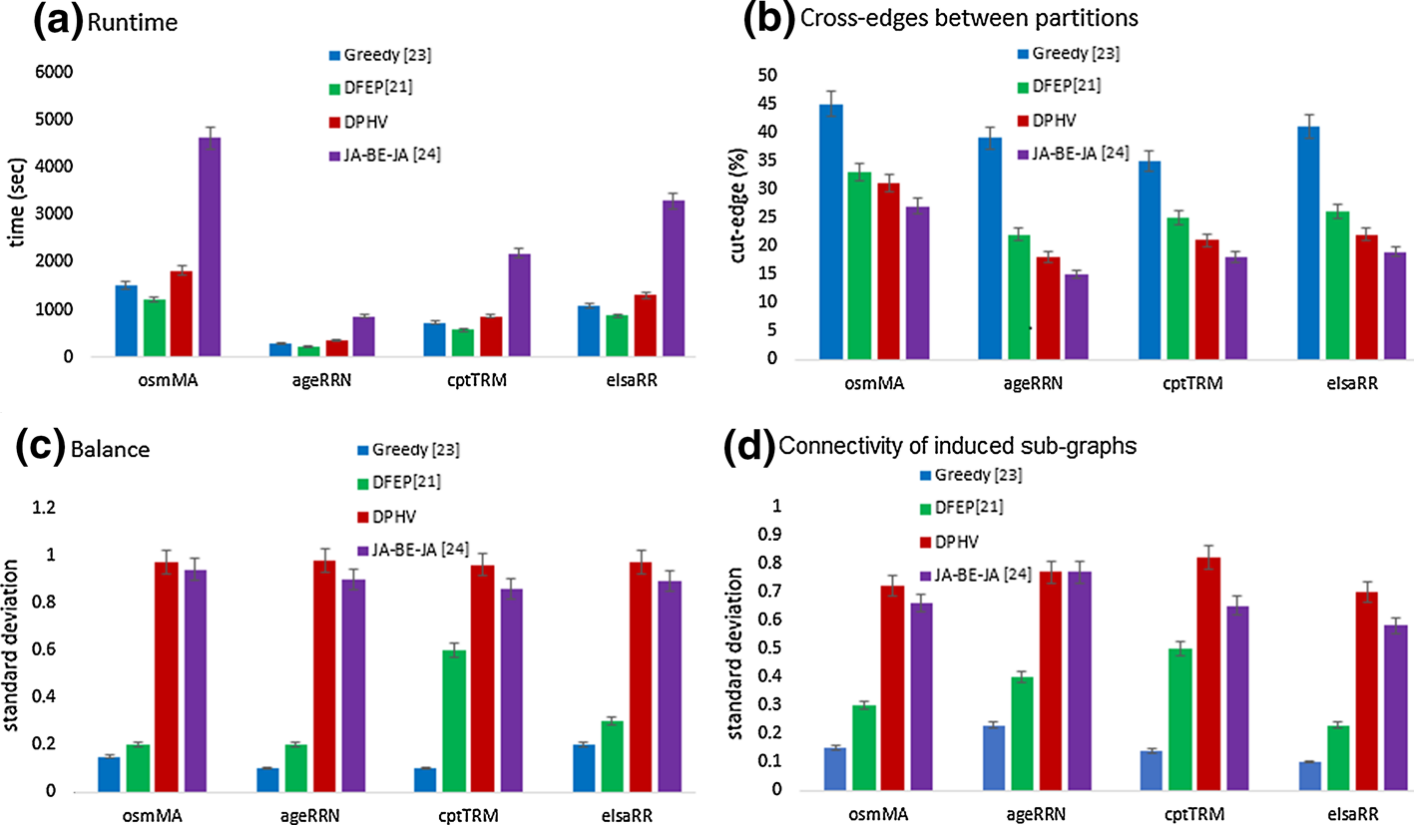
Comparison between DPHV, DFEP [21], JA-BE-JA [24] and Greedy [23] (k=100)

We compare our approach with others parallel and distributed algorithms: DFEP [21], JA-BE-JA [24] and Greedy [23]. Figure 16 shows the experimental results of graph algorithms on the datasets. In terms of velocity, Greedy [23] and DFED [21] outperform the performance of our algorithm. But DPHV presents better results of cut-edges compared to Greedy and DFED. The cut-edges with JA-BE-JA [24] are much better than ours.

In terms of partition balancing, our approach presents the best results compared to other algorithms. Also, our algorithm presents partitions whose sub-graphs induced in each partition are strongly connected.

Table 3 shows the gain ratio of our partitioning method compared to other partitioning techniques.

**Table 3:** DPHV performance ratio compared to DFEP [21], JA-BE-JA [24] and Greedy [23]

	DPHV vs. Greedy [23]	DPHV vs. DFEP [21]	DPHV vs. JA-BE-JA [24]
Time	− 1.2	− 1.5	*+ 2.53*
Cut-edges	*+ 1.74*	*+ 1.15*	− 0.86
Balance	*+ 7.08*	*+ 2.98*	*+ 1.08*
Connectivity	*+ 4.85*	*+ 2.1*	*+ 1.13*

In terms of velocity, DPHV is 1.2 times slower than Greedy and 1.5 times slower than DFEP [21]. But it is 2.53 times faster than JA-BE-JA [24]. The communication costs (cut-edges) proposed by our approach are 1.74 times reduced compared to that of Greedy [23] and 1.15 times reduced compared to DFEP [21]. On the other hand, the cut-edges of JA-BE-JA [24] optimize these cuts by 0.86 times than that of our algorithm. In terms of load balancing, our results are remarkable compared to the others. Our technique offers partitions whose weights are 7 times more balanced compared to Greedy [23] and 3 times more balanced compared to DFEP [21]. Similarly, in terms of connectivity, the partitions proposed by DPHV are strongly connected, this means that we maintain the topology of the original graph.

Besides, our algorithm outperforms all the distributed algorithms in terms of connectivity of the induced sub-graphs and offers the best performance. Our results are almost 5 times better than those of Greedy [23], 2 times better than those proposed by DFEP [21] and finally 1.13 times better than the connectivity of the induced sub-graphs resulting from the partitioning of JA-BE-JA [24].

### Discussions

Despite the fact that DPHV is efficient compared to other distributed algorithms [21, 23, 24], there are some limitations regarding the conceptual model, the programming paradigm and the applicability:

1. Node storage capacity [15]: DPHV is based on the Spark architecture and makes extensive use of certain functions of the Spark API. These functions are optimized for in-memory computations. In the case of big graphs, it becomes expensive to store the graph on RAM memory. So to maintain a good performance of our algorithm, we will have to allocate additional RAM memory.
2. Hardware failure [45]: DPHV partitioning task is completely decentralized to the slave nodes of the cluster. In the conceptual model of DPHV, when a node fails or is unavailable, the partitioning switches from a k-partition to a $(k-k')$-partition where $k'$ is the number of unavailable nodes. This therefore affects the weight of the sub-graphs contained in the unavailable nodes. Therefore, balancing the weights of the partitions cannot be guaranteed. Also, the cut-edges will be affected, resulting in a considerable communication cost.
3. Physical partitioning of graphs [14]: The partitioning logic of DPHV is based on the degree of the vertices in order to have balanced partitions. In the definition of a big graph, the vertices and edges store a large amount of data. Unfortunately, this is not taken into account in the DPHV partitioning logic. Therefore, despite the fact that the weights of the sub-graphs are balanced, they do not store the same amount of information.

In terms of complexity, our approach has advantages over DFEP [21]. Because in each iteration, it exchanges fewer vertices than DFEP [21]. As a result, DPHV optimizes the use of hardware resources such as ram memory, CPU and network processor.

Compared to our approach, JA-BE-JA [24] provides more optimal partitions because it is based on simulated annealing [39]. But very expensive in terms of time complexity and hardware resources.

Similarly, Greedy [23] outperforms our algorithm, but it is adapted for high-performance computing with single machine. This is very expensive in terms of hardware resources because it requires a very costly supercomputer [5].

## Conclusion and future work

In this paper, we have proposed a concrete formalism of the k-partition problem on big graphs. Moreover, we proposed a comparative study and a roadmap of partitioning algorithms. We introduced DPHV, a distributed k-partition algorithm based on a master-slaves architecture. In terms of velocity, DPHV is very fast and efficiently partitions a big graph into k sub-graphs of nearly similar weight while optimizing the number of cut-edges of the partition. DPHV also retains the topology of the original graph in a distributed architecture. The conceptual model of our framework is based on a coordinator and a set of partitioners. Experimental results have shown that our partitioning technique guarantees two fundamental properties : (1) the balancing of partition weights and (2) the preservation of the original graph topology in a distributed environment.

For future work, we are interested in expanding the scope of this work in the fight against covid-19. In particular by applying DPHV for the partitioning of large-scale community network, we can perform the propagation analysis and prediction of the COVID-19 by using all-shortest paths algorithms[4]. In addition, we are interested in proposing an extended version of the DPHV algorithm which sorts the vertices of the graph in such a way that the data contained in the vertices are consistent when Hadoop [45] physically splits the graph file.

## Data Availability

The data used for this study are available at :  https://snap.stanford.edu/data/index.html  https://projects.info.unamur.be/vibes/mutants-equiv.html  http://download.geofabrik.de/

## Abbreviations

DPHV: Distributed placement of hub-vertices
DFEP: Distributed funding-edge partitioning
IoT: Internet of things
CAP: Consistency availability partition
EPGM: Extended property graph model
CRUD: Create read update delete
NoSQL: Not only SQL
HDFS: Hadoop distributed file system
SNAP: Stanford network analysis platform
BSP: Bulk synchronous parallel
JOSM: Java open street map
OSM: Open street map

## Footnotes

1 https://www.grid5000.fr

2 https://snap.stanford.edu/

3 https://projects.info.unamur.be/vibes/mutants-equiv.html

4 http://download.geofabrik.de/
